# Supplementary material for: Multi‐dataset identification of innovative feature genes and molecular mechanisms in keratoconus
Source: J Cell Mol Med. 2024 Sep 19;28(18):e70079. doi: 10.1111/jcmm.70079 (PMC11412914; doi:10.1111/jcmm.70079)
Supplement: Supplementary file 2 — Material S2. [file JCMM-28-e70079-s002.zip › 7.GSEA analysis/5.RNF217-AS1/KEGG_OLFACTORY_TRANSDUCTION.html]

Details for gene set KEGG\_OLFACTORY\_TRANSDUCTION[GSEA]

|  || Dataset | RNF217-AS1.group.cls#h\_versus\_l |
| Phenotype | group.cls#h\_versus\_l |
| Upregulated in class | h |
| GeneSet | KEGG\_OLFACTORY\_TRANSDUCTION |
| Enrichment Score (ES) | 0.24987608 |
| Normalized Enrichment Score (NES) | 0.82907856 |
| Nominal p-value | 0.955102 |
| FDR q-value | 1.0 |
| FWER p-Value | 1.0 |
Table: GSEA Results Summary

  

Fig 1: Enrichment plot: KEGG\_OLFACTORY\_TRANSDUCTION      
 Profile of the Running ES Score & Positions of GeneSet Members on the Rank Ordered List

  

| SYMBOL | TITLE | RANK IN GENE LIST | RANK METRIC SCORE | RUNNING ES | CORE ENRICHMENT || 1 | OR10A6 | na | 112 | 0.713 | 0.0068 | Yes |
| 2 | OR52E8 | na | 149 | 0.689 | 0.0156 | Yes |
| 3 | OR10A3 | na | 220 | 0.641 | 0.0226 | Yes |
| 4 | OR52H1 | na | 252 | 0.616 | 0.0304 | Yes |
| 5 | OR51A7 | na | 287 | 0.603 | 0.0380 | Yes |
| 6 | OR8J1 | na | 368 | 0.569 | 0.0437 | Yes |
| 7 | OR8B4 | na | 383 | 0.564 | 0.0513 | Yes |
| 8 | OR13F1 | na | 428 | 0.549 | 0.0578 | Yes |
| 9 | OR2L2 | na | 521 | 0.526 | 0.0626 | Yes |
| 10 | OR6C1 | na | 560 | 0.517 | 0.0688 | Yes |
| 11 | OR2A42 | na | 580 | 0.514 | 0.0755 | Yes |
| 12 | OR1F1 | na | 762 | 0.476 | 0.0770 | Yes |
| 13 | OR10G7 | na | 854 | 0.462 | 0.0809 | Yes |
| 14 | OR1K1 | na | 1076 | 0.432 | 0.0806 | Yes |
| 15 | OR4F6 | na | 1147 | 0.423 | 0.0846 | Yes |
| 16 | OR4K17 | na | 1220 | 0.417 | 0.0884 | Yes |
| 17 | OR10J1 | na | 1228 | 0.416 | 0.0941 | Yes |
| 18 | OR8D1 | na | 1238 | 0.415 | 0.0997 | Yes |
| 19 | OR1L8 | na | 1313 | 0.408 | 0.1033 | Yes |
| 20 | CALML6 | na | 1514 | 0.391 | 0.1030 | Yes |
| 21 | OR10G8 | na | 1531 | 0.389 | 0.1081 | Yes |
| 22 | OR4K15 | na | 1571 | 0.385 | 0.1124 | Yes |
| 23 | OR5B3 | na | 1816 | 0.365 | 0.1105 | Yes |
| 24 | OR13C3 | na | 1879 | 0.360 | 0.1138 | Yes |
| 25 | OR6N1 | na | 1880 | 0.360 | 0.1189 | Yes |
| 26 | OR5H2 | na | 1890 | 0.359 | 0.1237 | Yes |
| 27 | OR4C3 | na | 1962 | 0.353 | 0.1266 | Yes |
| 28 | OR2A12 | na | 2166 | 0.336 | 0.1255 | Yes |
| 29 | OR2A2 | na | 2215 | 0.332 | 0.1288 | Yes |
| 30 | OR8S1 | na | 2263 | 0.330 | 0.1321 | Yes |
| 31 | OR4F15 | na | 2317 | 0.326 | 0.1352 | Yes |
| 32 | OR2AT4 | na | 2336 | 0.325 | 0.1392 | Yes |
| 33 | OR5C1 | na | 2372 | 0.323 | 0.1428 | Yes |
| 34 | OR10K2 | na | 2438 | 0.320 | 0.1455 | Yes |
| 35 | OR2M4 | na | 2454 | 0.319 | 0.1495 | Yes |
| 36 | OR51F2 | na | 2507 | 0.316 | 0.1525 | Yes |
| 37 | OR10S1 | na | 2579 | 0.312 | 0.1549 | Yes |
| 38 | OR6C6 | na | 2658 | 0.307 | 0.1569 | Yes |
| 39 | OR51Q1 | na | 2716 | 0.305 | 0.1596 | Yes |
| 40 | OR5M8 | na | 2785 | 0.301 | 0.1619 | Yes |
| 41 | OR9K2 | na | 2816 | 0.300 | 0.1653 | Yes |
| 42 | OR52K2 | na | 2871 | 0.297 | 0.1679 | Yes |
| 43 | OR7D2 | na | 2932 | 0.294 | 0.1703 | Yes |
| 44 | OR52N1 | na | 3010 | 0.291 | 0.1722 | Yes |
| 45 | OR5AR1 | na | 3025 | 0.290 | 0.1759 | Yes |
| 46 | OR51E1 | na | 3029 | 0.290 | 0.1799 | Yes |
| 47 | OR6V1 | na | 3046 | 0.289 | 0.1835 | Yes |
| 48 | OR11L1 | na | 3144 | 0.285 | 0.1848 | Yes |
| 49 | OR2G6 | na | 3197 | 0.282 | 0.1872 | Yes |
| 50 | OR2A14 | na | 3298 | 0.279 | 0.1883 | Yes |
| 51 | OR6C70 | na | 3485 | 0.272 | 0.1867 | Yes |
| 52 | CAMK2A | na | 3603 | 0.267 | 0.1871 | Yes |
| 53 | OR5AC2 | na | 3642 | 0.266 | 0.1898 | Yes |
| 54 | OR2M3 | na | 3658 | 0.265 | 0.1931 | Yes |
| 55 | OR5AP2 | na | 3728 | 0.262 | 0.1948 | Yes |
| 56 | OR2AG1 | na | 3789 | 0.260 | 0.1967 | Yes |
| 57 | OR5T1 | na | 3799 | 0.260 | 0.2002 | Yes |
| 58 | OR52E6 | na | 3807 | 0.259 | 0.2036 | Yes |
| 59 | OR4S2 | na | 3868 | 0.258 | 0.2055 | Yes |
| 60 | OR5AS1 | na | 3919 | 0.256 | 0.2077 | Yes |
| 61 | OR56A1 | na | 3999 | 0.252 | 0.2090 | Yes |
| 62 | OR14C36 | na | 4087 | 0.249 | 0.2100 | Yes |
| 63 | OR1N2 | na | 4191 | 0.246 | 0.2105 | Yes |
| 64 | OR10X1 | na | 4224 | 0.244 | 0.2130 | Yes |
| 65 | OR5D13 | na | 4246 | 0.243 | 0.2158 | Yes |
| 66 | OR2L3 | na | 4319 | 0.240 | 0.2171 | Yes |
| 67 | OR5M1 | na | 4335 | 0.240 | 0.2201 | Yes |
| 68 | OR10T2 | na | 4403 | 0.237 | 0.2215 | Yes |
| 69 | OR5B12 | na | 4440 | 0.236 | 0.2238 | Yes |
| 70 | OR2T10 | na | 4496 | 0.234 | 0.2255 | Yes |
| 71 | OR14A16 | na | 4536 | 0.232 | 0.2277 | Yes |
| 72 | OR1L1 | na | 4636 | 0.229 | 0.2280 | Yes |
| 73 | OR1D2 | na | 4665 | 0.228 | 0.2305 | Yes |
| 74 | OR11G2 | na | 4724 | 0.227 | 0.2320 | Yes |
| 75 | OR9Q1 | na | 4940 | 0.219 | 0.2288 | Yes |
| 76 | OR5B17 | na | 4943 | 0.219 | 0.2319 | Yes |
| 77 | OR13C9 | na | 4990 | 0.217 | 0.2336 | Yes |
| 78 | CAMK2B | na | 5000 | 0.217 | 0.2364 | Yes |
| 79 | OR6C75 | na | 5005 | 0.217 | 0.2394 | Yes |
| 80 | OR1C1 | na | 5038 | 0.216 | 0.2415 | Yes |
| 81 | OR13G1 | na | 5167 | 0.211 | 0.2408 | Yes |
| 82 | OR8K1 | na | 5168 | 0.211 | 0.2437 | Yes |
| 83 | OR52B6 | na | 5260 | 0.208 | 0.2440 | Yes |
| 84 | OR2T8 | na | 5351 | 0.205 | 0.2443 | Yes |
| 85 | OR5AN1 | na | 5364 | 0.204 | 0.2469 | Yes |
| 86 | OR8B12 | na | 5795 | 0.190 | 0.2370 | Yes |
| 87 | OR1D5 | na | 5866 | 0.187 | 0.2377 | Yes |
| 88 | OR1J1 | na | 5921 | 0.186 | 0.2387 | Yes |
| 89 | OR8D2 | na | 5964 | 0.184 | 0.2401 | Yes |
| 90 | OR2M7 | na | 6097 | 0.180 | 0.2388 | Yes |
| 91 | OR4P4 | na | 6172 | 0.178 | 0.2392 | Yes |
| 92 | OR52N5 | na | 6176 | 0.178 | 0.2416 | Yes |
| 93 | OR1N1 | na | 6178 | 0.178 | 0.2441 | Yes |
| 94 | CNGA4 | na | 6192 | 0.177 | 0.2462 | Yes |
| 95 | OR51B6 | na | 6349 | 0.173 | 0.2441 | Yes |
| 96 | OR2T34 | na | 6436 | 0.170 | 0.2441 | Yes |
| 97 | OR56A5 | na | 6479 | 0.169 | 0.2452 | Yes |
| 98 | OR5M9 | na | 6514 | 0.167 | 0.2466 | Yes |
| 99 | OR5T3 | na | 6564 | 0.166 | 0.2475 | Yes |
| 100 | OR2H2 | na | 6565 | 0.166 | 0.2499 | Yes |
| 101 | OR13D1 | na | 6767 | 0.160 | 0.2463 | No |
| 102 | OR5D16 | na | 7049 | 0.151 | 0.2403 | No |
| 103 | OR4Q3 | na | 7269 | 0.145 | 0.2359 | No |
| 104 | OR5L2 | na | 7315 | 0.144 | 0.2367 | No |
| 105 | OR9Q2 | na | 7531 | 0.139 | 0.2324 | No |
| 106 | OR7A5 | na | 7544 | 0.139 | 0.2340 | No |
| 107 | OR14I1 | na | 7598 | 0.138 | 0.2344 | No |
| 108 | OR2T11 | na | 7665 | 0.136 | 0.2344 | No |
| 109 | OR2W3 | na | 7818 | 0.132 | 0.2319 | No |
| 110 | OR6M1 | na | 7957 | 0.129 | 0.2297 | No |
| 111 | OR51L1 | na | 8020 | 0.127 | 0.2297 | No |
| 112 | OR13C2 | na | 8027 | 0.127 | 0.2313 | No |
| 113 | OR2T2 | na | 8037 | 0.127 | 0.2328 | No |
| 114 | PRKG1 | na | 8062 | 0.126 | 0.2339 | No |
| 115 | OR51M1 | na | 8064 | 0.126 | 0.2357 | No |
| 116 | OR10A4 | na | 8250 | 0.122 | 0.2320 | No |
| 117 | OR2T27 | na | 8275 | 0.121 | 0.2330 | No |
| 118 | OR2L13 | na | 8314 | 0.121 | 0.2336 | No |
| 119 | OR9A4 | na | 8331 | 0.120 | 0.2349 | No |
| 120 | OR2AK2 | na | 8810 | 0.109 | 0.2225 | No |
| 121 | OR10G3 | na | 8914 | 0.107 | 0.2210 | No |
| 122 | OR52D1 | na | 9051 | 0.104 | 0.2185 | No |
| 123 | OR4C46 | na | 9121 | 0.103 | 0.2180 | No |
| 124 | OR5A1 | na | 9207 | 0.101 | 0.2169 | No |
| 125 | OR1B1 | na | 9247 | 0.100 | 0.2172 | No |
| 126 | OR6K3 | na | 9270 | 0.099 | 0.2180 | No |
| 127 | OR56B4 | na | 9282 | 0.099 | 0.2191 | No |
| 128 | OR6S1 | na | 9470 | 0.096 | 0.2150 | No |
| 129 | OR3A2 | na | 9543 | 0.094 | 0.2142 | No |
| 130 | OR4X2 | na | 9626 | 0.093 | 0.2132 | No |
| 131 | OR4A15 | na | 9677 | 0.092 | 0.2130 | No |
| 132 | OR4C11 | na | 9839 | 0.089 | 0.2096 | No |
| 133 | OR11H4 | na | 9895 | 0.088 | 0.2092 | No |
| 134 | OR6X1 | na | 9907 | 0.088 | 0.2102 | No |
| 135 | OR1A1 | na | 10000 | 0.087 | 0.2087 | No |
| 136 | OR52K1 | na | 10298 | 0.081 | 0.2012 | No |
| 137 | OR5D14 | na | 10312 | 0.081 | 0.2020 | No |
| 138 | OR6K6 | na | 10476 | 0.078 | 0.1984 | No |
| 139 | OR5K2 | na | 10484 | 0.078 | 0.1993 | No |
| 140 | OR4N2 | na | 10554 | 0.077 | 0.1983 | No |
| 141 | PRKG2 | na | 10584 | 0.077 | 0.1986 | No |
| 142 | OR13H1 | na | 10704 | 0.075 | 0.1962 | No |
| 143 | OR51V1 | na | 10816 | 0.073 | 0.1940 | No |
| 144 | OR4M1 | na | 11371 | 0.065 | 0.1788 | No |
| 145 | OR10A5 | na | 11497 | 0.063 | 0.1760 | No |
| 146 | OR1J4 | na | 11528 | 0.062 | 0.1760 | No |
| 147 | OR10R2 | na | 11546 | 0.062 | 0.1764 | No |
| 148 | ARRB2 | na | 11624 | 0.061 | 0.1750 | No |
| 149 | OR5P3 | na | 11687 | 0.060 | 0.1741 | No |
| 150 | OR4C12 | na | 11701 | 0.059 | 0.1745 | No |
| 151 | OR10G9 | na | 11801 | 0.058 | 0.1725 | No |
| 152 | OR2S2 | na | 11831 | 0.058 | 0.1725 | No |
| 153 | OR52B2 | na | 11929 | 0.056 | 0.1704 | No |
| 154 | OR2V2 | na | 11967 | 0.056 | 0.1701 | No |
| 155 | OR2T4 | na | 12117 | 0.054 | 0.1666 | No |
| 156 | OR4D1 | na | 12140 | 0.053 | 0.1667 | No |
| 157 | OR8J3 | na | 12269 | 0.052 | 0.1637 | No |
| 158 | OR6A2 | na | 12418 | 0.050 | 0.1601 | No |
| 159 | OR8K3 | na | 12559 | 0.048 | 0.1567 | No |
| 160 | OR2AG2 | na | 12581 | 0.048 | 0.1568 | No |
| 161 | OR4N5 | na | 12648 | 0.047 | 0.1555 | No |
| 162 | OR10AD1 | na | 12762 | 0.045 | 0.1529 | No |
| 163 | OR9G4 | na | 12867 | 0.044 | 0.1504 | No |
| 164 | OR56B1 | na | 12891 | 0.044 | 0.1504 | No |
| 165 | OR51A4 | na | 12904 | 0.043 | 0.1507 | No |
| 166 | OR4X1 | na | 13005 | 0.042 | 0.1483 | No |
| 167 | OR10H2 | na | 13027 | 0.042 | 0.1483 | No |
| 168 | OR10AG1 | na | 13038 | 0.042 | 0.1486 | No |
| 169 | OR8G1 | na | 13359 | 0.038 | 0.1399 | No |
| 170 | OR2AE1 | na | 13364 | 0.038 | 0.1403 | No |
| 171 | OR6B2 | na | 13541 | 0.036 | 0.1357 | No |
| 172 | OR2G2 | na | 13753 | 0.033 | 0.1300 | No |
| 173 | OR2J2 | na | 13803 | 0.033 | 0.1290 | No |
| 174 | OR4A16 | na | 13840 | 0.032 | 0.1284 | No |
| 175 | CALM2 | na | 13884 | 0.032 | 0.1276 | No |
| 176 | OR52N2 | na | 14064 | 0.030 | 0.1228 | No |
| 177 | OR4K2 | na | 14123 | 0.029 | 0.1216 | No |
| 178 | GUCA1B | na | 14200 | 0.028 | 0.1198 | No |
| 179 | OR4K1 | na | 14234 | 0.028 | 0.1192 | No |
| 180 | OR4K5 | na | 14322 | 0.027 | 0.1170 | No |
| 181 | CNGB1 | na | 14323 | 0.027 | 0.1174 | No |
| 182 | OR52J3 | na | 14368 | 0.026 | 0.1165 | No |
| 183 | OR10H5 | na | 14402 | 0.026 | 0.1159 | No |
| 184 | OR6F1 | na | 14569 | 0.024 | 0.1114 | No |
| 185 | OR10Z1 | na | 14795 | 0.021 | 0.1052 | No |
| 186 | OR2B3 | na | 14799 | 0.021 | 0.1054 | No |
| 187 | OR6B1 | na | 14818 | 0.021 | 0.1052 | No |
| 188 | OR1Q1 | na | 14934 | 0.020 | 0.1021 | No |
| 189 | OR1L6 | na | 14973 | 0.019 | 0.1013 | No |
| 190 | OR2H1 | na | 15061 | 0.018 | 0.0990 | No |
| 191 | OR51B5 | na | 15128 | 0.018 | 0.0973 | No |
| 192 | OR5I1 | na | 15162 | 0.017 | 0.0966 | No |
| 193 | OR6B3 | na | 15191 | 0.017 | 0.0960 | No |
| 194 | OR10J3 | na | 15232 | 0.017 | 0.0951 | No |
| 195 | OR8I2 | na | 15374 | 0.015 | 0.0912 | No |
| 196 | OR1A2 | na | 15403 | 0.015 | 0.0906 | No |
| 197 | CALML5 | na | 15459 | 0.014 | 0.0892 | No |
| 198 | OR4S1 | na | 15593 | 0.013 | 0.0855 | No |
| 199 | CLCA4 | na | 15600 | 0.013 | 0.0855 | No |
| 200 | OR5V1 | na | 15673 | 0.012 | 0.0836 | No |
| 201 | OR2G3 | na | 15790 | 0.011 | 0.0804 | No |
| 202 | OR2C3 | na | 15795 | 0.011 | 0.0804 | No |
| 203 | OR6K2 | na | 15921 | 0.010 | 0.0769 | No |
| 204 | CAMK2G | na | 16021 | 0.008 | 0.0742 | No |
| 205 | OR56A3 | na | 16039 | 0.008 | 0.0738 | No |
| 206 | OR51B4 | na | 16088 | 0.008 | 0.0725 | No |
| 207 | OR6C4 | na | 16214 | 0.007 | 0.0690 | No |
| 208 | OR8A1 | na | 16476 | 0.004 | 0.0614 | No |
| 209 | OR52E4 | na | 16547 | 0.003 | 0.0594 | No |
| 210 | OR2W1 | na | 16751 | 0.001 | 0.0535 | No |
| 211 | CLCA2 | na | 16918 | -0.001 | 0.0487 | No |
| 212 | OR5AK2 | na | 17064 | -0.002 | 0.0445 | No |
| 213 | CALML3 | na | 17310 | -0.005 | 0.0375 | No |
| 214 | OR4A5 | na | 17545 | -0.007 | 0.0307 | No |
| 215 | GUCY2D | na | 17844 | -0.009 | 0.0222 | No |
| 216 | OR51I1 | na | 17960 | -0.010 | 0.0190 | No |
| 217 | OR8D4 | na | 18040 | -0.011 | 0.0169 | No |
| 218 | OR2D3 | na | 18059 | -0.011 | 0.0165 | No |
| 219 | OR2Z1 | na | 18080 | -0.012 | 0.0161 | No |
| 220 | CALM3 | na | 18318 | -0.014 | 0.0094 | No |
| 221 | GNAL | na | 18447 | -0.015 | 0.0059 | No |
| 222 | OR9G1 | na | 18496 | -0.015 | 0.0047 | No |
| 223 | OR51F1 | na | 19023 | -0.020 | -0.0103 | No |
| 224 | OR7G2 | na | 19667 | -0.027 | -0.0287 | No |
| 225 | OR1S1 | na | 19795 | -0.028 | -0.0320 | No |
| 226 | OR5T2 | na | 19849 | -0.028 | -0.0331 | No |
| 227 | OR5J2 | na | 19875 | -0.029 | -0.0334 | No |
| 228 | OR4A47 | na | 19996 | -0.030 | -0.0365 | No |
| 229 | CAMK2D | na | 20347 | -0.033 | -0.0462 | No |
| 230 | OR7C2 | na | 20600 | -0.036 | -0.0530 | No |
| 231 | OR10G2 | na | 20665 | -0.037 | -0.0544 | No |
| 232 | OR4N4 | na | 21335 | -0.043 | -0.0732 | No |
| 233 | OR3A1 | na | 21896 | -0.050 | -0.0888 | No |
| 234 | OR2T6 | na | 22375 | -0.055 | -0.1020 | No |
| 235 | OR2M5 | na | 22445 | -0.055 | -0.1032 | No |
| 236 | OR5B21 | na | 22477 | -0.056 | -0.1033 | No |
| 237 | OR52L1 | na | 22495 | -0.056 | -0.1030 | No |
| 238 | OR4C6 | na | 22509 | -0.056 | -0.1026 | No |
| 239 | ADCY3 | na | 22794 | -0.059 | -0.1100 | No |
| 240 | PRKACA | na | 23066 | -0.063 | -0.1170 | No |
| 241 | OR11A1 | na | 23131 | -0.063 | -0.1180 | No |
| 242 | OR52W1 | na | 23603 | -0.070 | -0.1307 | No |
| 243 | OR5L1 | na | 24057 | -0.076 | -0.1428 | No |
| 244 | OR10K1 | na | 24169 | -0.078 | -0.1449 | No |
| 245 | CLCA1 | na | 24290 | -0.079 | -0.1473 | No |
| 246 | OR8K5 | na | 24516 | -0.083 | -0.1527 | No |
| 247 | OR51G1 | na | 24527 | -0.083 | -0.1518 | No |
| 248 | OR10A2 | na | 24544 | -0.083 | -0.1511 | No |
| 249 | OR1I1 | na | 24610 | -0.084 | -0.1518 | No |
| 250 | OR10P1 | na | 25251 | -0.094 | -0.1691 | No |
| 251 | OR9I1 | na | 25300 | -0.095 | -0.1691 | No |
| 252 | OR2L8 | na | 25415 | -0.097 | -0.1711 | No |
| 253 | OR2D2 | na | 25461 | -0.098 | -0.1710 | No |
| 254 | OR6N2 | na | 25628 | -0.100 | -0.1744 | No |
| 255 | OR1L4 | na | 25684 | -0.101 | -0.1746 | No |
| 256 | OR8G5 | na | 25849 | -0.104 | -0.1779 | No |
| 257 | OR5D18 | na | 25908 | -0.105 | -0.1780 | No |
| 258 | OR7E24 | na | 25923 | -0.106 | -0.1770 | No |
| 259 | OR4K13 | na | 25990 | -0.107 | -0.1774 | No |
| 260 | OR6C76 | na | 26016 | -0.107 | -0.1766 | No |
| 261 | OR6C68 | na | 26250 | -0.111 | -0.1818 | No |
| 262 | PDE1C | na | 26305 | -0.113 | -0.1818 | No |
| 263 | OR6T1 | na | 26342 | -0.113 | -0.1812 | No |
| 264 | OR10J5 | na | 26410 | -0.115 | -0.1815 | No |
| 265 | PRKACB | na | 26457 | -0.116 | -0.1812 | No |
| 266 | CALM1 | na | 26557 | -0.117 | -0.1824 | No |
| 267 | OR4C15 | na | 26727 | -0.120 | -0.1857 | No |
| 268 | OR51S1 | na | 26908 | -0.124 | -0.1891 | No |
| 269 | OR6C74 | na | 26926 | -0.124 | -0.1879 | No |
| 270 | OR6C2 | na | 27028 | -0.126 | -0.1890 | No |
| 271 | OR13J1 | na | 27476 | -0.136 | -0.2001 | No |
| 272 | OR12D2 | na | 27598 | -0.139 | -0.2017 | No |
| 273 | OR6C65 | na | 27677 | -0.140 | -0.2020 | No |
| 274 | OR10C1 | na | 27833 | -0.145 | -0.2044 | No |
| 275 | OR5M10 | na | 27840 | -0.145 | -0.2025 | No |
| 276 | OR5AU1 | na | 27963 | -0.148 | -0.2040 | No |
| 277 | OR2A25 | na | 28009 | -0.149 | -0.2032 | No |
| 278 | OR2B2 | na | 28173 | -0.153 | -0.2058 | No |
| 279 | OR9A2 | na | 28267 | -0.155 | -0.2063 | No |
| 280 | OR4D9 | na | 28295 | -0.156 | -0.2049 | No |
| 281 | OR4C45 | na | 28332 | -0.157 | -0.2037 | No |
| 282 | OR5K1 | na | 28379 | -0.158 | -0.2028 | No |
| 283 | OR11H6 | na | 28536 | -0.163 | -0.2050 | No |
| 284 | OR52A5 | na | 28625 | -0.165 | -0.2052 | No |
| 285 | OR10A7 | na | 28684 | -0.168 | -0.2045 | No |
| 286 | OR52N4 | na | 28685 | -0.168 | -0.2022 | No |
| 287 | OR12D3 | na | 28737 | -0.169 | -0.2013 | No |
| 288 | OR5P2 | na | 28758 | -0.170 | -0.1994 | No |
| 289 | OR13C8 | na | 28807 | -0.171 | -0.1984 | No |
| 290 | OR4D10 | na | 29053 | -0.178 | -0.2030 | No |
| 291 | OR7D4 | na | 29145 | -0.180 | -0.2031 | No |
| 292 | OR51I2 | na | 29157 | -0.181 | -0.2009 | No |
| 293 | OR52R1 | na | 29192 | -0.181 | -0.1993 | No |
| 294 | OR51A2 | na | 29248 | -0.183 | -0.1983 | No |
| 295 | OR51B2 | na | 29282 | -0.184 | -0.1967 | No |
| 296 | OR4D2 | na | 29387 | -0.188 | -0.1970 | No |
| 297 | OR10G4 | na | 29402 | -0.188 | -0.1948 | No |
| 298 | OR4D6 | na | 29487 | -0.191 | -0.1945 | No |
| 299 | OR10H4 | na | 29510 | -0.191 | -0.1924 | No |
| 300 | OR52M1 | na | 29547 | -0.192 | -0.1908 | No |
| 301 | OR2A7 | na | 29605 | -0.194 | -0.1897 | No |
| 302 | OR3A3 | na | 29623 | -0.195 | -0.1874 | No |
| 303 | OR5F1 | na | 29657 | -0.196 | -0.1856 | No |
| 304 | OR2K2 | na | 29685 | -0.196 | -0.1836 | No |
| 305 | OR8U1 | na | 29695 | -0.197 | -0.1811 | No |
| 306 | OR4B1 | na | 29729 | -0.198 | -0.1792 | No |
| 307 | OR6C3 | na | 29777 | -0.199 | -0.1778 | No |
| 308 | OR7G3 | na | 29822 | -0.201 | -0.1762 | No |
| 309 | OR51E2 | na | 29909 | -0.203 | -0.1758 | No |
| 310 | OR14J1 | na | 29935 | -0.204 | -0.1737 | No |
| 311 | OR51G2 | na | 30025 | -0.207 | -0.1733 | No |
| 312 | OR8B8 | na | 30096 | -0.210 | -0.1724 | No |
| 313 | OR1J2 | na | 30236 | -0.214 | -0.1734 | No |
| 314 | OR52B4 | na | 30312 | -0.217 | -0.1725 | No |
| 315 | OR10Q1 | na | 30378 | -0.219 | -0.1713 | No |
| 316 | OR2F1 | na | 30432 | -0.221 | -0.1697 | No |
| 317 | OR4D11 | na | 30510 | -0.224 | -0.1688 | No |
| 318 | OR5A2 | na | 30622 | -0.228 | -0.1688 | No |
| 319 | OR5M11 | na | 30668 | -0.229 | -0.1669 | No |
| 320 | OR10V1 | na | 30772 | -0.233 | -0.1666 | No |
| 321 | OR7G1 | na | 30824 | -0.235 | -0.1647 | No |
| 322 | CNGA3 | na | 30895 | -0.238 | -0.1634 | No |
| 323 | OR4C13 | na | 30917 | -0.238 | -0.1606 | No |
| 324 | OR7A10 | na | 31054 | -0.244 | -0.1611 | No |
| 325 | OR56A4 | na | 31291 | -0.254 | -0.1644 | No |
| 326 | OR2A5 | na | 31333 | -0.256 | -0.1620 | No |
| 327 | PRKX | na | 31362 | -0.257 | -0.1591 | No |
| 328 | OR5M3 | na | 31543 | -0.266 | -0.1606 | No |
| 329 | OR2T12 | na | 31570 | -0.267 | -0.1576 | No |
| 330 | OR7C1 | na | 31588 | -0.268 | -0.1543 | No |
| 331 | OR4L1 | na | 31629 | -0.270 | -0.1516 | No |
| 332 | OR2J3 | na | 31655 | -0.272 | -0.1485 | No |
| 333 | OR10H1 | na | 31993 | -0.291 | -0.1542 | No |
| 334 | OR2B11 | na | 32183 | -0.302 | -0.1554 | No |
| 335 | OR6Q1 | na | 32229 | -0.305 | -0.1524 | No |
| 336 | OR4E2 | na | 32233 | -0.305 | -0.1482 | No |
| 337 | OR2T33 | na | 32285 | -0.308 | -0.1453 | No |
| 338 | OR4C16 | na | 32424 | -0.319 | -0.1448 | No |
| 339 | GUCA1A | na | 32468 | -0.322 | -0.1415 | No |
| 340 | OR1L3 | na | 32475 | -0.323 | -0.1371 | No |
| 341 | OR13A1 | na | 32523 | -0.326 | -0.1338 | No |
| 342 | OR1E1 | na | 32584 | -0.330 | -0.1309 | No |
| 343 | OR7A17 | na | 32609 | -0.332 | -0.1269 | No |
| 344 | OR1S2 | na | 32655 | -0.335 | -0.1235 | No |
| 345 | OR2B6 | na | 32657 | -0.335 | -0.1188 | No |
| 346 | OR2T1 | na | 32665 | -0.335 | -0.1142 | No |
| 347 | OR52I1 | na | 32713 | -0.339 | -0.1108 | No |
| 348 | OR8H3 | na | 32722 | -0.339 | -0.1062 | No |
| 349 | OR1G1 | na | 32799 | -0.345 | -0.1035 | No |
| 350 | OR13C4 | na | 32878 | -0.351 | -0.1008 | No |
| 351 | OR13C5 | na | 33010 | -0.361 | -0.0995 | No |
| 352 | OR6Y1 | na | 33032 | -0.363 | -0.0950 | No |
| 353 | PRKACG | na | 33174 | -0.374 | -0.0938 | No |
| 354 | OR2T5 | na | 33207 | -0.377 | -0.0894 | No |
| 355 | OR52E2 | na | 33360 | -0.391 | -0.0883 | No |
| 356 | OR8U8 | na | 33395 | -0.394 | -0.0837 | No |
| 357 | OR2Y1 | na | 33524 | -0.409 | -0.0816 | No |
| 358 | OR2F2 | na | 33622 | -0.422 | -0.0785 | No |
| 359 | OR4K14 | na | 33659 | -0.426 | -0.0735 | No |
| 360 | OR51D1 | na | 33771 | -0.443 | -0.0704 | No |
| 361 | OR51T1 | na | 33810 | -0.449 | -0.0652 | No |
| 362 | OR52A1 | na | 33871 | -0.458 | -0.0604 | No |
| 363 | OR4D5 | na | 34040 | -0.490 | -0.0584 | No |
| 364 | PDC | na | 34114 | -0.505 | -0.0534 | No |
| 365 | GUCA1C | na | 34160 | -0.517 | -0.0473 | No |
| 366 | OR2M2 | na | 34215 | -0.531 | -0.0414 | No |
| 367 | OR2C1 | na | 34232 | -0.536 | -0.0343 | No |
| 368 | OR1M1 | na | 34238 | -0.538 | -0.0268 | No |
| 369 | OR8H2 | na | 34419 | -0.608 | -0.0234 | No |
| 370 | OR52I2 | na | 34424 | -0.610 | -0.0149 | No |
| 371 | OR8H1 | na | 34545 | -0.682 | -0.0087 | No |
| 372 | OR5H6 | na | 34705 | -0.989 | 0.0007 | No |
Table: GSEA details [plain text format]

  

Fig 2: KEGG\_OLFACTORY\_TRANSDUCTION      
 Blue-Pink O' Gram in the Space of the Analyzed GeneSet

  

Fig 3: KEGG\_OLFACTORY\_TRANSDUCTION: Random ES distribution      
 Gene set null distribution of ES for **KEGG\_OLFACTORY\_TRANSDUCTION**
